# Evidence that Northern Pioneering Pines with Tuberculate Mycorrhizae are Unaffected by Varying Soil Nitrogen Levels

**DOI:** 10.1007/s00248-012-0076-0

**Published:** 2012-06-08

**Authors:** William Kenneth Chapman, Leslie Paul

**Affiliations:** 1 Research Section, BC Ministry of Forests LNRO, 200-640 Borland Street, Williams Lake, BC Canada; 2 Swedish University of Agricultural Sciences, Box 7070, 750 07 Uppsala, Sweden

## Abstract

Tuberculate mycorrhizae on *Pinus contorta* (lodgepole pine) have previously been shown to reduce acetylene, but an outstanding question has been to what degree these structures could meet the nitrogen requirements of the tree. We compared the growth, tissue nitrogen contents, and stable nitrogen isotope ratios of *P*. *contorta* growing in gravel pits to the same species growing on adjacent intact soil. Trees growing in severely nitrogen deficient gravel pits had virtually identical growth rates and tissue nitrogen contents to those growing on intact soil that had nitrogen levels typical for the area. δ^15^N values for trees in the gravel pits were substantially lower than δ^15^N values for trees on intact soil, and isotope ratios in vegetation were lower than the isotope ratios of the soil. The form of soil nitrogen in the gravel pits was almost exclusively nitrate, while ammonium predominated in the intact soil. Discrimination against ^15^N during plant uptake of soil nitrate in the highly N-deficient soil should be weak or nonexistent. Therefore, the low δ^15^N in the gravel pit trees suggests that trees growing in gravel pits were using another nitrogen source in addition to the soil. Precipitation-borne nitrogen in the study area is extremely low. In conjunction with our other work, these findings strongly suggests that *P*. *contorta* and its microbial symbionts or associates fix nitrogen in sufficient amounts to sustain vigorous tree growth on the most nitrogen-deficient soils.

## Introduction

*Pinus contorta* (lodgepole pine) can be found growing well throughout British Columbia on the most nitrogen-deficient soils such as unreclaimed gravel pits and road cuts. Nitrogen levels in lodgepole pine, unlike in other species such as hybrid spruce, are sometimes poorly linked to soil nitrogen levels [1]. Some possible explanations for this include, for example, that *P*. *contorta*, through its physiology or mycorrhizal symbionts, is able to more effectively access and take up soil nitrogen than other tree species or that it has relatively higher levels of associative [2] or even symbiotic nitrogen fixation.

Our previous work [3] has suggested that *P*. *contorta*, in conjunction with one of its tuberculate mycorrhizal (mycorrhizae that consist of a tight cluster of branched short roots encased in mycelium and usually a peridium to form a tubercle) symbionts, *Suillus tomentosus*, and identified bacterial species are capable of reducing acetylene at high rates, while non-tuberculate mycorrhizae are not. We have found abundant tuberculate mycorrhizae in low nitrogen forests [4], but have had difficulty answering the question of whether or not nitrogen fixation in these structures can supply a substantial portion of the tree’s nitrogen needs. While we have demonstrated in situ high levels of acetylene reduction activity in *S*. *tomentosus* tuberculate mycorrhizae on *P*. *contorta* [5], that nitrogen-fixing bacteria can be isolated from within tuberculate mycorrhizae [3], that tuberculate mycorrhizae are abundant in some types of sub-boreal forests [4], and that tuberculate mycorrhizae contain a unique hemoglobin-like molecule [3], none of these constitutes proof that *P*. *contorta* is able to meet its nitrogen requirements through current nitrogen fixation on or around roots.

One simple and direct way to establish whether or not pine is able to meet its nitrogen nutritional requirements with limited soil-based nitrogen source is to examine pine in the most nitrogen-deficient environments to see if growth or nitrogen contents are restricted. For non-nitrogen-fixing forest plants, there is a reliable relationship between soil and tissue nitrogen levels and growth [6].

In this work, we compare tissue nitrogen contents and growth rates of *P*. *contorta* in undisturbed forest soil (secondary succession) to trees in highly disturbed and extremely nitrogen-deficient soils (gravel pits—primary succession), where atmospheric nitrogen inputs are very low. The purpose is to see if *P*. *contorta* has the capacity to meet its nitrogen growth requirements in the absence of significant soil or precipitation sources of nitrogen. In addition, nitrogen isotope analysis is used in this study to try to understand which nitrogen pools are being accessed by *P*. *contorta* growing on extremely nitrogen-deficient soils.

The basis for using nitrogen isotope natural abundance to determine source pools of nitrogen is the variation in the concentration of ^15^N in different nitrogen pools caused by discrimination against the heavier isotope during certain biological processes. Symbiotic N-fixation weakly discriminates against ^15^N [7, 8] or not at all [9]. Decomposing soil organic matter becomes enriched in ^15^N [10] because microbes are presumably slower to take up ^15^N and nitrification plus, either leaching of ^15^N-depleted nitrate or denitrification of the same can concentrate ^15^N in the remaining pool [11]. Ammonium volatilization can also concentrate ^15^N in soil [7]. Soil mineral N that derives from mineralized organic matter could be enriched in ^15^N [12]. Also, there appears to be a relationship between nitrogen uptake and the size of the nitrogen pool. Where a large proportion of a pool of nitrogen is taken up by a plant community relative to the replenishment rate of the pool, there is little discrimination because ^15^N rapidly concentrates as a result of plant uptake and so is absorbed at higher rates before plant uptake is satiated. Where the pool of nitrogen is large and quickly replenished, there may be considerable discrimination because even during periods of high plant uptake, the lighter isotope remains in greater abundance relative to the heavier isotope [6, 7]. Ectomycorrhizal fungi accumulate ^15^N, and there is depletion of ^15^N in plants where nitrogen has passed through ectomycorrhizal fungi [6]. However, when nitrate is the primary nitrogen source, ectomycorrhizal plants differ little from source N or non-mycorrhizal plants in nitrogen isotope ratio [13]. For some plants, there seems to be variable small discrimination when source levels of nitrate are high and a small to no discrimination when they are low [7, 14]. Climate also has an effect on foliar δ^15^N [9], but in this study, both treatments per site are paired in close proximity, and climate effects should not be an issue. Though there are a large number of interacting factors that affect nitrogen isotope ratios, natural abundance levels can sometimes help clarify nitrogen source.

## Materials and Methods

### Site Selection

Six gravel pits located in south-central British Columbia were utilized in this study. The pits are located within a 100 km by 50 km area defined by 52 ° 27′ 41.86″ N, 52 ° 02′ 10.45″ N, 123 ° 10′ 24.42″ W, and 121 ° 50′ 05.78″ W. The forests in this area are predominantly *P*. *contorta* with *Picea glauca* x *Picea engelmannii* (hybrid spruce) in wetter soils and in more mature stands. Some *Pseudotsuga menziesii* (Douglas-fir) was also present around the edges of some of the gravel pits. In British Columbia, these forest types are called Sub-Boreal Spruce and Sub-Boreal Pine Spruce [15]. All soils in this study are gravelly in texture with weak profile development, while the gravel pits do not have soil horizons, but rather the “soil” consists of gravelly parent material. Normal annual precipitation for 1971 to 2000 at the Williams Lake Airport (which is central to the study area) is 0.30 m/a rainfall, 0.15 m/a snowfall (as liquid) for total annual precipitation of 0.45 m/a as liquid. The average yearly temperature is 4.2 °C

### Whole Tree Sampling

Three to four whole (roots, shoots, and boles) *P*. *contorta* trees between 1 and 2 m in height were excavated in each of the gravel pits (pit trees) and from adjacent to the gravel pits (intact soil trees) at each of six sites (approximately six trees per site, 38 trees in total). It was difficult to find enough trees of the same age and size to destructively sample (that we could access with an excavator), which is why the number of trees varies per site. Because we had to take what was available, by chance, the sample ended up being skewed in terms of size with the pit trees being larger than the intact soil trees. Intact soil trees were taken from areas where the forest had been cut during gravel pit development, but the overburden (≈topsoil) had not been stripped away (soil intact). The trees in the gravel pit were growing on bare gravel without any other nitrogen-fixing species within at least 20 m (usually much more than 20 m, nitrogen-fixing *Shepherdia canadensis* (L.) Nutt. and *Alnus* spp. occurred infrequently in the pits). Whole root systems were excavated carefully to try to keep short roots (mycorrhizae) as intact as possible using a combination of machine digging to undercut the roots and gentle hand digging to encourage the gravel and sand to fall free of the roots. All trees excavated were fully colonized by mycorrhizae with abundant tuberculate mycorrhizae, as well as many other kinds of mycorrhizae.

### δ^15^N and Total Nitrogen

The trees were air dried and separated into needles, branches, boles, and roots. The tissue was ground, and δ^15^N and total percent nitrogen were determined by the University of Waterloo Environmental Isotope Laboratory. Samples were analysed on a Delta Plus Continuous Flow Stable Isotope Ratio Mass Spectrometer (Thermo Finnigan, Bremen, Germany) coupled to a Carlo Erba Elemental Analyzer (CHNS-O EA1108; Italy). Stable isotope ratios are expressed as delta (δ) and are measures of a “per mil” (‰), or parts per thousand of difference between the isotope ratio of a sample and that of atmospheric air for nitrogen. The equation describing the ratio from Mariotti [16] is:


By convention, the air ratios are set to a value of 0 ‰, and this is the standard against which other materials are measured. Results are generally corrected to nitrogen standards IAEA-N1 and IAEA-N2. The error for clean ball-milled standard material is ± −0.3 ‰ for nitrogen. This error can be expected to increase depending on the homogeneity, type, and amount of sample used in analysis.

### Tree Growth Rates

Trees for growth rate determination were selected to be between 1.3 and 1.5 m tall. They were chosen to fall within a size range rather than by age because we reasoned that trees in the gravel pits might be slow to establish, and we wanted to compare growth rates of trees with approximately equal canopy sizes rather than trees of the same age. Tree age was determined by counting branch whorls, with random checks of the accuracy of whorl, counting by cutting trees off at the root collar, and counting tree rings. Two of three checks agreed exactly with whorl counting, and one check differed by 1 year. Trees for growth rate determination were chosen to be free of disease, forks, or damage of any kind. They also had to be completely open to sunlight on three sides (no shadowing or overtopping by other trees). Trees sampled outside the gravel pits grew in intact topsoil, while the trees in the gravel pit grew in bare gravel without any nitrogen fixing species within 20 m. Tree height, leader length, and root collar diameter were measured.

### Soil Nitrogen Levels

Six mineral soil samples were collected to a depth of 0.2 m from each gravel pit and bulked into two samples (to get a measure of variability of the sampling method). Three samples were taken from the soil intact areas at each site and bulked into one sample. The samples were air dried and sieved to separate the <0.002 m fraction. The <0.002 m fraction was analysed for total C, NH_4_-N, and NO_3_-N by the BC Ministry of Forests and Range Research Laboratory in Victoria BC. Isotope ratios were determined by the same lab described above for vegetation analyses. There were forest floors present in the soil intact areas, but forest floors were not initially sampled. We know from other work that forest floors in the area are typically around 1 % nitrogen [19], and we determined nitrogen isotope ratios on forest floor samples taken from a trial located 1.24 km from the Skulow pit site.

### Precipitation-Borne Nitrogen

For rainfall measurements, two Cole Parmer #03319-10 rainfall collection gauges were installed at each gravel pit. The gauges were put out July 4, 2008, and collected rainfall was taken in monthly until October 20, 2008. The Palmer site gauges were stolen. The precipitation samples were stored in sealed glass bottles at 3 °C and kept for stable isotope N and nitrogen content analyses; however, it turned out that nitrogen levels were too low to permit the isotope analysis. The water was analyzed directly for inorganic N (nitrate-N and ammonium-N) using an OI-Analytical “Alpkem FSIV” automated chemistry analyzer. Total N was determined using an autoclave-heated alkaline persulfate digestion followed by determination of produced nitrate on the FSIV analyzer. Snowfall samples were collected in March 2008, and stored frozen until they could be analysed for stable nitrogen isotopes and nitrogen contents. These samples also had nitrogen levels too low for the isotope analysis to be conducted (University of Waterloo Environmental Isotope Laboratory, direct communication).

An alternative method for precipitation sampling was also used during the rainfall collection period. One or two ion exchange resin precipitation samplers were installed at each site. Two went missing, and one was damaged which left seven samplers that were extracted. The collectors were designed and extracted as per the method of J.A. Simmons [17]. Each collector contained 0.035 kg of MB-150 mixed bed ion exchange resin (Amberlite brand) and was extracted with two 0.0002 m^3^ washes of 2 N KCl. The extracts were filtered through a 0.45 u membrane syringe filter and immediately analyzed for ammonium-N and nitrate(+nitrite)-N using an OI Analytical, Alpkem automated chemistry analyzer. A separate filtered subsample was digested using alkaline persulfate in tightly sealed head space vials, heated for 30 min in an autoclave at 123 °C. A ratio of four parts persulfate to one part sample was used to ensure complete oxidation of nitrogen in the presence of high chloride levels. A volume of 0.0002 m^3^ of the alkaline persulfate reagent was autoclaved for later use in preparing calibration standards. The oxidized samples were analyzed for nitrate-N using the Alpkem analyzer. Several glycine-N standards in 2 N KCl, treated the same way as the unknown samples, were analyzed simultaneously to confirm full recovery. Standards used for calibrating the Alpkem analyzer were prepared in 2 N KCl and diluted one part to four with the autoclaved alkaline persulfate reagent. This ensured that the matrix of the standards was identical to the samples and also eliminated the need to account for a dilution factor.

## Results

### δ^15^N and Total Nitrogen in Trees

The nitrogen content and delta values for the various parts of the trees are summarized in Table 1. The nitrogen contents of the gravel pit and intact soil trees are virtually identical by tissue type, except for the roots (which are only slightly different). The δ^15^N values are typically about 1.5 times lower in the gravel pit trees. *t* Tests indicate the differences between the percent nitrogen values, which are not statistically significant (*p* (two tailed) = 0.67), while the differences between the delta values are highly significant (*p* (two tailed) < 0.001).

**Table 1:** Tree nitrogen content and isotope ratios by tissue type and mean

	Number	Percent nitrogen		δ^15^N	
		Mean	SD	Mean	SD
Boles in soil	19	0.47	0.07	−2.93	1.52
Boles in pit	19	0.49	0.09	−4.37	1.37
Branches in soil	19	0.58	0.08	−3.30	1.69
Branches in pit	19	0.58	0.08	−4.69	1.34
Needles in soil		1.22	0.15	−2.09	1.61
Needles in pit	19	1.25	0.19	−3.42	1.32
Roots in soil	19	0.32	0.13	−1.04	1.30
Roots in pit	19	0.25	0.08	−1.69	0.94
Forest mean	19	0.65	0.11	−2.34	1.53
Pit mean		0.64	0.11	−3.54	1.24

Overall, there was no correlation between needle δ values for nitrogen and needle mass (e.g., needle mass and δ^15^N *r* ^2^ = 0.04, significance of *F* of 0.20), and there was no correlation between needle δ and needle mass for the intact soil trees (e.g., forest bole mass and δ^15^N *r* ^2^ = 0.05, significance of *F* of 0.30). However, for the pit trees, needle δ^15^N and needle mass had a positive correlation with an *r* ^2^ = 0.22 and a significance of *F* of 0.04. The correlation between pit tree bole mass and bole δ^15^N was also positive with an *r* ^2^ = 0.21 and significance of *F* of 0.05. Only the pit trees show any relationship between measures of mass and δ^15^N.

### Tree Growth Rates

The growth data for the trees are summarized in Table 2. The average age, height, and leader of the sampled trees are virtually identical. The trees were selected to be in the same height range, so the purpose of the sampling was to compare leader lengths by treatment. The tree ages turned out to be, on average, identical, and the only difference in growth was the root collar diameter, which is significantly (*p* (two tailed) = 0.01) greater in the pit trees.

**Table 2:** Growth comparison of pine trees by treatment

	Mean	SD	*P* (*T* < *t*) two-tail
Height for soil (m)	1.412	0.056	0.96
Height for pit (m)	1.412	0.069	
Leader for soil (m)	0.307	0.011	0.6
Leader for pit (m)	0.320	0.082	
Root collar for soil (m)	0.0266	0.0055	0.01
Root collar for pit (m)	0.0304	0.0055	
Age for soil (Whorls)	7.7	2.6	0.9
Age for pit (Whorls)	7.7	1.6	

*n* = 30

The pit trees and the intact soil trees for the destructive whole tree sampling were not equal in size. The pit trees ended up being on average 1.9 times bigger by bole mass.

### Soil Nitrogen Levels

Table 3 shows the analyses for the soil nitrogen content. The intact soil had approximately sixfold higher total nitrogen (*p* (two tailed) = 0.006) and carbon levels than the substrate in gravel pits. The gravel pits have a much higher proportion of soil nitrogen as nitrate than the intact soil (*p* (two tailed) = 0.03). In five out of six sites, ammonium levels were not detectable in the pit samples, but in the intact soil, ammonium was always quite considerably the larger of the two nitrogen forms measured. We did not compare forest floor nitrogen levels as the gravel pits did not have organic forest floors.

**Table 3:** Soil carbon and nitrogen levels

Site	Pit or soil	Total C	NH4-N	NO3-N	Total N
		(%)	PPM	PPM	(%)
Mountain House	Pit	0.27	<0.01	0.33	0.01
	Soil	2.50	3.16	0.13	0.12
Skulow	Pit	0.85	<0.01	0.41	0.03
	Soil	0.96	0.97	0.28	0.06
Miocene	Pit	0.31	0.05	0.74	0.02
	Soil	1.28	2.13	<0.01	0.06
Whitestone	Pit	0.24	<0.01	0.30	0.01
	Soil	3.54	3.83	0.13	0.16
Anah	Pit	0.25	<0.01	0.18	0.01
	Soil	2.14	3.17	0.05	0.08
Palmer	Pit	0.19	<0.01	0.42	0.01
	Soil	2.01	3.40	0.08	0.08
Pit mean		0.351	0.046	0.397	0.015
Plus/minus		0.26	0.03	0.23	0.01
Soil mean		2.072	2.779	0.136	0.094
SD		0.92	1.05	0.09	0.04

Each pit measure is the mean of two bulked samples consisting of three samples each, and the soil sample measures are taken on one bulked sample consisting of three samples

### Soil Isotope Ratios

The soil δ^15^N are shown in Table 4. The pit samples had significantly (*p* (two tailed) = 0.04) lower (pit average = 1.74, soil average = 2.73) δ^15^N values. The average δ^15^N for the two forest floor samples was 1.04.

**Table 4:** Soil δ^15^N levels

	δ^15^N	
	Pit	Forest
Mountain House	0.64	2.08
Skulow	0.16	1.97
Palmer	2.12	2.57
Whitestone	3.64	3.15
Miocene	2.72	4.17
Anah	1.18	2.41
Average	1.74	2.73
SD	1.32	0.82
Skulow Forest Floor		0.96
		1.11
Average		1.04

### Precipitation-Borne Nitrogen

The average total nitrogen content in rain (less two obviously contaminated samples) was 2.3^−7^ kg N L^−1^ (SD = 6.2^−8^ kg N L^−1^). The average total nitrogen content in snowfall was 1.7^−7^ kg N L^−1^of snow as water (SD = 8.0^−8^ kg N L^−1^). Based on the Environment Canada normal precipitation values presented in the methods, this equates to annual deposition rates of 6.9^−5^ kg N m^−2^ in rainfall and 2.7^−5^ kg N m^−2^ in snowfall, for a total of 9.6^−5^ kg N m^−2^ total nitrogen. This agrees closely with estimates of precipitation-borne nitrogen of 9.0^−5^ kg N m^−2^a^−1^ from a nearby area [18]. For the ion resin samples, one had anomalously high N content and was discolored (the discoloration could have been due to contamination from bird or other animal droppings) and so was disregarded. Based on six ion resin samples and the Environment Canada precipitation data, the mean annual deposition rate was 3.1^−4^ kg N m^−2^ (SD = 4.0^−5^ kg N m^−2^).

## Discussion

In this trial, we found that the tissue nitrogen contents and growth rates of *P*. *contorta* are unaffected by dramatic differences in soil nitrogen levels. The trees in pits and undisturbed soil were growing in close proximity to each other, with little environmental difference other than the soil; there are no other major nitrogen-fixing species in the vicinity of the sampled trees, and in the remote and industry-free study area, atmospheric inputs of nitrogen are very low and unlikely to contribute much to nitrogen sufficiency [22]. Other studies confirm that *P*. *contorta* growing in central British Columbia are indifferent to large differences in soil nitrogen levels [1, 18]. *P*. *contorta*'s indifference to soil nitrogen levels begs an explanation. Our work to date demonstrates that high rates of nitrogen fixation take place in *P*. *contorta* tuberculate mycorrhizae, but an outstanding question was whether or not *P*. *contorta* could meet its nitrogen nutritional requirements on a nitrogen-deficient soil. The combination of gravelly parent material for soil and low atmospheric inputs make the gravel pits used in this study site the most nitrogen limited environments we could find, yet the *P*. *contorta* in the gravel pits had growth rates that are typical for the area. This is very strong evidence that pine is able to meet its nitrogen nutritional requirements without a soil nitrogen source.

While we feel confident that this work unequivocally demonstrates *P*. *contorta*'s indifference to varying soil nitrogen levels, we used isotope analysis to try to determine the nitrogen pool that the trees in this study are accessing. Presented below are two different interpretations of the isotope data.

There are a large number of fractionating steps for ^15^N [9, 20] in the nitrogen cycle and it is difficult to account for all the different potential discrimination points to arrive at a convincing accounting of how isotope ratios will vary [21] even, as in this case, when comparing two treatments using the same species growing in close proximity to each other. A simplification of the complex situation is that ^15^N is only slightly discriminated against during biological fixation, which means that the δ^15^N of recently fixed nitrogen should be close to that of the atmosphere (atmospheric inputs are discounted in this case because they are so low and too low to measure isotope ratios). At various points in the nitrogen cycle, ^15^N may be concentrated as, for example, in soil during plant uptake, in mycorrhizae by mycorrhizal fungi, in various plant tissues during internal cycling, and in decomposing organic matter by saprotrophs. As a consequence of the concentration of ^15^N in the tissue of mycorrhizal fungi or to a lesser degree in the soil during direct plant uptake, the tissue of mycorrhizal plants tend to be depleted in ^15^N over the source. Though these effects shift the concentration of ^15^N in the various pools in a system, they do not change the isotope ratio of the system as a whole. Ultimately, ^15^N is concentrated in the system as a whole only by nitrification/denitrification, leaching of organic molecules or ammonium loss (less important in forest soil) [23], all of which favor the lighter isotope. All of the concentrating processes progress over time. In this study, we had an older intact soil where nitrogen had been fractionated for at least many decades and a very young soil in the early stages of accreting nitrogen from recent fixation and deposition, with little time for fractionation. Since each nitrogen pool measured in the pit system in this study had a smaller proportion of ^15^N than its counterpoint in the intact soil system, it is logical to conclude that the pit system as a whole has a higher proportion of recently fixed nitrogen than the soil system. It is possible that some not specifically measured pool, like the pool of nitrogen within mycorrhizal fungi, might have been significantly enriched in ^15^N and so have skewed all other pools in the pit. However, some of the mycelial ^15^N in the mycorrhizal fungus pool would have been accounted for in the soil sample, and much of the ^15^N concentrated in mycorrhizae themselves would have been accounted for in the root sample. It is extremely improbable that there would have been enough ^15^N concentrated in unsampled mycorrhizal fungus to offset the depletion in the entire rest of the system, including the soil. There were no other nitrogen-fixing plant species in this study, so any recently fixed nitrogen was either coming from free living bacteria, which is very unlikely in such a low energy system (and the likelihood of which was not supported by the very low soil nitrogen levels), from associative fixation around the pine roots or from symbiotic fixation in tuberculate mycorrhizae on the pine roots.

To approach the isotope data in another way, the nitrogen level of the soil in the pits was in the order of sixfold lower than in the intact soil and extremely deficient [24]. Therefore, there would be little chance for concentration of ^15^N in the pit soil pool by discrimination against ^15^N during plant uptake as soil nitrogen would deplete quickly and both isotopes should be entirely taken up [13, 20]. The δ^15^N values of plants growing in such a nitrogen-deficient soil should closely approach soil values unless mycorrhizal fungi were sequestering large amounts of ^15^N [6]. In the pit, almost all of the nitrogen was in nitrate form, and some work reports little or no discrimination against ^15^N during uptake where soil nitrogen is present predominantly as nitrate [7, 14]. This lack of discrimination during nitrate uptake seems to be especially true when uptake is high relative to supply as would particularly have been the case in the pit portion of this study. The lack of discrimination against nitrate ^15^N also seems to hold true for ectomycorrhizal species [13]. In Högberg et al. [13], it is suggested that the reason for the difference in discrimination behavior for nitrate versus ammonium is that the presences of a heavier nitrogen isotope in ammonium results in a much greater proportional increase in the mass of the ammonium ion than it would in a nitrate ion. This means that uptake or transformation by mycorrhizal fungi or any biological process should have only weak discrimination, if any, against ^15^N incorporated in nitrate.

It has been observed by several others that there is a widespread trend for plant tissue δ^15^N to be lower with lower soil nitrogen contents [9]. At face value, this could be all that is occurring in this study. Two explanations are commonly offered for low tissue ^15^N [9] with low soil nitrogen. The first is that where soil nitrogen supply is high relative to demand, through a variety of processes and over time, ^15^N becomes concentrated in the soil [7], and the resulting higher soil δ^15^N values are ultimately reflected in the plants. Where nitrogen levels are generally low, there is no concentration of ^15^N because all available soil nitrogen is consumed, and so no concentration can occur. The second explanation is based on the observation that mycorrhizal fungi accumulate ^15^N. It is suggested that mycorrhizae would play a bigger role in nitrogen uptake in lower nitrogen soils, and so the lower the nitrogen content of the soil, the more ^15^N would accumulate in mycorrhizal fungi and the lower the δ^15^N of the plant tissue.

In this study we determined δ^15^N by plant part, and the roots of the pit trees also had lower δ^15^N values than the roots of the intact soil trees, so since total tree nitrogen levels were so similar, this finding is contrary to the likelihood that pit trees accumulated a greater proportion of ^15^N in mycorrhizae. The roots of both treatments had the highest δ^15^N values of all the tissue pools sampled, which is consistent with the mycorrhizal fungus accumulation theory, but they were still lower than (or in the case of the pit sample almost equal to) the soil δ^15^N values. If concentration of ^15^N within mycorrhizae was the major cause of ^15^N depletion (over soil values) in the upper plant tissue, then the roots (with mycorrhizae) should have had higher δ^15^N values than the soil. The roots can be broken into two pools; secondary roots which would also have received ^15^N-depleted nitrogen from mycorrhizae and the mycorrhizal roots where much of the concentrated ^15^N would have accumulated. Taken together, these two root pools still constitute a much smaller nitrogen pool than the rest of plant, and since a large proportion of the ^15^N depleted from the nitrogenous compounds that went to the tops should have accumulated in the roots as a whole, then the roots as a whole should have had δ^15^N values that were noticeably higher than the soil, which they did not. By contrast, if the plant nitrogen is largely coming from nitrogen fixation taking place in the mycorrhizae, then the baseline δ^15^N would be a combination of soil and atmosphere values, and the roots would be enriched by mycorrhizal discrimination to some level above the unknown (but lower than soil), combined base value. This remains a possible scenario. However, this argument cannot be taken too far because it was very hard to harvest roots with all of their mycorrhizae intact, even though we were very careful. Also, the roots were superficially contaminated with soil, which may have had some effect on δ^15^N values.

While the repeated observations of accumulation of ^15^N in mycorrhizal fungi do make a robust case [9] that ^15^N accumulation in mycorrhizal fungi does normally occur, it is still possible that another factor could also contribute to the trend of generally lower δ^15^N in aerial tissue with declining soil nitrogen. Since recently fixed nitrogen tends to have lower δ^15^N values than soil [21], the result of an increased use of recently fixed nitrogen by plants growing on nitrogen poor soil would, in most instances, be lower isotope ratios than would result from plants using soil nitrogen alone. It seems possible that this could be the cause of the lower tissue δ^15^N in this study and it might be worth exploring to see if this could be a factor in other cases where δ^15^N is lower in low nitrogen soils.

Since free living and associative fixation typically amount to only a small proportions of symbiotic fixation per unit area [25], and since soil nitrogen levels are extremely low in the gravel pits aside from in the trees, the most logical conclusion is that nitrogen is being fixed within or in close proximity to pine roots and moving directly into the pine trees. The possibility of nitrogen fixation in tuberculate mycorrhizae on *P*. *contorta* is in keeping with our previously published work which has shown high levels of acetylene reduction in situ on *P*. *contorta* tuberculate mycorrhizae (fungal symbiont—*S*. *tomentosus*) [5]. We have also demonstrated significant quantities of *S*. *tomentosus* tuberculate mycorrhizae in *P*. *contorta* forests [1], isolated nitrogen-fixing bacteria from within tuberculate mycorrhizae, and identified a hemoglobin-like molecule from the tuberculate mycorrhizae [1]. This accumulating evidence strongly suggests that *P*. *contorta*, in association with its fungal and bacterial symbionts, is a major nitrogen-fixing species.

In the comparison of growth rates, it could be argued that, by selecting trees of the same size, we might have inadvertently selected genetically slower growing trees in the intact soil and genetically faster growing trees in the gravel pits to get them to be in the same size class. Both in the gravel pits and beside the gravels pits, we ended up measuring virtually the entire population of trees that met our criteria. A wide range of trees sizes and ages were available, so a sample from within a narrow size range should have represented a cross-section of faster growing younger trees and slower growing older trees if the trees were growing at different rates. It turned out that the trees were very similar in age and growth rates, which means that there was little genetically derived variation in performance to be found. Therefore, we feel confident to say that, based on root collar diameter and leader length, the trees in the gravel pits are growing at the same rate or faster than the trees in the intact soil.

For trees growing in the gravel pits (only), the larger the tree the higher the δ^15^N. This suggests that older trees could be fixing less nitrogen and concentrating ^15^N by internal N cycling [26, 27] or other mechanisms. In previous work [5], we found higher levels of acetylene reduction activity in younger stands. However, many things could be going on. Perhaps as trees get larger, soil nitrogen pools are also increasing with typically higher δ^15^N values, and the trees become less dependent on recent fixation and mycorrhizae. Larger trees also have more extensive root systems to intercept precipitation. Since the gravel pit trees sampled for isotope ratio were by chance larger than the sampled intact soil trees, if the difference in tree size was linked to the cause of the differences in δ^15^N values between the two treatments, then it would be expected that the gravel pit trees would have had higher δ^15^N values. The difference in δ^15^N values was opposite in direction from what would have been expected if size had been linked to the cause of the difference between treatments.

Many of the *P*. *contorta* forests in the interior of British Columbia are thought to transition to spruce and are called sub-boreal spruce or sub-boreal pine spruce [15] forests. The fire return frequency is so high in parts of the sub-boreal pine spruce forest that some of these forests seem to be perpetually in pine sere. Many soils in the study area, even outside the gravel pits, are poorly developed and nitrogen poor from the cold, dry climate, and/or frequent fire volatilization of nutrients and sometimes, as in this study, from coarse textured soil with low nutrient holding capacity. We contend that *P*. *contorta* with its microbial symbionts acts a pioneer species in both primary and secondary succession in the forests of British Columbia, and the widespread abundance of *P*. *contorta* in BC is the result of the perpetually low nitrogen capital of many of the forest soils in the area.

Initiatives to offset the effects of mountain pine beetle on wood supply in British Columbia include contemplation to fertilize large areas of pine forest with nitrogen. There could still be positive growth effects from providing easily accessed nitrogen to pine trees; but on the scale of good ways to invest money, we concur with previous work that shows that the economics of fertilizing *P*. *contorta*'s has to be examined carefully [28]. We feel that pine could be used more effectively in managed forests if its pioneering nature is better exploited. The beneficial effect of pines on the growth of other species has long been observed [29]. The ability of pine, in conjunction with its symbionts, to fix nitrogen could explain this effect. Northern pines, such as *P*. *contorta*, and their root symbionts also have great potential for reclaiming highly disturbed areas such as oil sands and mine spoils in forested northern Canadian ecosystems. *P*. *contorta* has been widely and successfully used in the Central Interior of British Columbia to rehabilitate highly disturbed areas associated with logging.

If nitrogen is ubiquitously fixed in boreal and temperate forests, then nitrogen fertilizer response in these systems may simply be a response to nitrogen that is energetically cheaper to the plant. A large, but less available, pool of nitrogen tied up in organic matter could have benefits that outweigh the advantages of a more readily available, but also more mobile mineral nitrogen pool. Understanding that nitrogen availability in northern forests is not necessarily limited by the capacity of the system to fix nitrogen should have great bearing on how northern forests are managed.

## Conclusion

*P*. *contorta* is able to meet all of its nitrogen requirements for normal growth when growing on the most nitrogen-deficient environment we could find. The distribution of naturally occurring nitrogen isotopes in this study is not inconsistent with the possibility that the trees were accessing recently fixed nitrogen. Based on our previous work which demonstrates high levels of acetylene reduction in tuberculate mycorrhizae in *P*. *contorta*, we conclude that the simplest and most likely explanation for comparable growth and nitrogen levels between trees growing on intact soil and those growing on very low nitrogen gravel is that this pine species, in conjunction with certain symbionts, is capable of fixing biologically significant quantities of nitrogen.
